# Investigation of Biocompatible PEO Coating Growth on cp-Ti with In Situ Spectroscopic Methods

**DOI:** 10.3390/ma15010009

**Published:** 2021-12-21

**Authors:** Veta Aubakirova, Ruzil Farrakhov, Arseniy Sharipov, Veronika Polyakova, Lyudmila Parfenova, Evgeny Parfenov

**Affiliations:** 1 Department of Electronic Engineering, Ufa State Aviation Technical University, 12 Karl Marx Street, 450008 Ufa, Russia; veta_mr@mail.ru (V.A.); frg1982@mail.ru (R.F.); arsenyarseny36728@gmail.com (A.S.); 2 Institute of Physics of Advanced Materials, Ufa State Aviation Technical University, 12 Karl Marx Street, 450008 Ufa, Russia; polyakova.vv@ugatu.su; 3 Department of General Chemistry, Ufa State Aviation Technical University, 12 Karl Marx Street, 450008 Ufa, Russia; luda_parfenova@mail.ru; 4 Department of Biomedical Engineering, Ufa State Aviation Technical University, 12 Karl Marx Street, 450008 Ufa, Russia

**Keywords:** functional biocompatible coating, plasma electrolytic oxidation, microdischarges, process diagnostics, optical emission spectroscopy, in-situ impedance spectroscopy

## Abstract

The problem of the optimization of properties for biocompatible coatings as functional materials requires in-depth understanding of the coating formation processes; this allows for precise manufacturing of new generation implantable devices. Plasma electrolytic oxidation (PEO) opens the possibility for the design of biomimetic surfaces for better biocompatibility of titanium materials. The pulsed bipolar PEO process of cp-Ti under voltage control was investigated using joint analysis of the surface characterization and by in situ methods of impedance spectroscopy and optical emission spectroscopy. Scanning electron microscopy, X-ray diffractometry, coating thickness, and roughness measurements were used to characterize the surface morphology evolution during the treatment for 5 min. In situ impedance spectroscopy facilitated the evaluation of the PEO process frequency response and proposed the underlying equivalent circuit where parameters were correlated with the coating layer properties. In situ optical emission spectroscopy helped to analyze the spectral line evolutions for the substrate material and electrolyte species and to justify a method to estimate the coating thickness via the relation of the spectral line intensities. As a result, the optimal treatment time was established as 2 min; this provides a 9–11 µm thick PEO coating with Ra 1 µm, 3–5% porosity, and containing 75% of anatase. The methods for in-situ spectral diagnostics of the coating thickness and roughness were justified so that the treatment time can be corrected online when the coating achieves the required properties.

## 1. Introduction

The world market of orthopedic implants is constantly growing due to the aging population [1]. The most preferred materials for reconstructive surgery in orthopedics are titanium alloys, which are used for the production of screws, fixation plates, scaffolds, and other internal devices [2]. However, titanium itself does not show bioactive properties, and commercially used alloys such as Ti–6Al–4V contain harmful alloying elements; therefore, the implant surface must be functionalized with a corrosion resistant and bioactive coating [3,4]. Otherwise, the surface will not properly interact with the osteoblast cells, and a foreign body reaction and inflammation would either slow the implant integration or cause implant rejection [5]. This complex problem in the area of functional materials can be solved by a proper design of the chemical and phase composition, and morphology of the coating [6]. The biomimetic approach appears to be the most fruitful decision of this problem [7,8].

Plasma electrolytic oxidation (PEO) is one of the promising methods for the biomimetic coating formation on the surface of light alloys including titanium [9,10]. This method affords obtaining a ceramic porous coating on Al, Ti, Zr, Mg, Nb, and other valve metals [11,12,13,14]; the coating properties can be controlled by the variation of the process parameters and electrolyte composition as well as through the realization of the process control techniques [15,16,17]. Such morphology provides a gradual change in the elasticity modulus from the metal substrate to the bone; this addresses the biomimetic requirements at the mechanical level. The porous morphology has a bone-like structure, therefore, it provides close to the natural conditions for the osteoblast attachment and contributes to the biomimetic requirements at the physical level [7]. By introducing bioactive species and elements such as hydroxyapatite, silver, silicon, magnesium, fluorine, and others, the PEO process can address the biomimetic requirements at the chemical level on all implantable materials such as Ti, Mg, and others [18,19,20,21]. As previously shown, cp-Ti surface biofunctionalization with a titania PEO coating and organic molecules appears to be more effective than the formation of Ca- and P- containing inorganic PEO coatings [22]. Therefore, this research focuses on a titania PEO coating for cp-Ti as a substrate for further modification with the bioactive organic molecules addressing the biomimetic requirements at the biological level [23].

The early stages of the PEO process predefine the quality of the resultant coating [24]. Therefore, an in-depth evaluation of the PEO process and resultant coatings during the treatment are crucial for the development of a high-quality biocompatible coating. Nowadays, investigations into the early stages of the Al [25], Mg [26,27], and Ti [28,29] PEO show new insights into the process mechanism. Due to the different electric regimes, not all results are comparable. PEO of Ti can be preformed in direct current (DC) [30], pulsed unipolar DC [31], and pulsed bipolar [32] regimes; all regimes can be realized either under current [33] or voltage control [34] and the combination of the regimes, and voltage ramping is also possible [35]. However, the question of the optimal PEO process duration and methods to understand in situ whether or not the process should be stopped is still open, especially for the treatment of titanium alloys under the voltage control. Previously, various diagnostic methods based on electrical and optical characteristics of the PEO process have been developed [36,37,38,39]. The in-depth evaluation of the PEO coatings via complex in situ electric diagnostics in both unipolar and bipolar regimes has recently been presented [40]. These methods include in situ impedance spectroscopy, which helps to justify the equivalent circuit of the system “metal-oxide-microdischarge-electrolyte” [41], and optical emission spectroscopy, which provides the emission intensities of the substrate and electrolyte species and facilitates estimation of the coating thickness [42].

Therefore, the aim of this research was to investigate the PEO coating growth on cp-Ti under the voltage control through the analysis of the surface morphology, coating thickness, roughness, and phase composition in comparison with the results of the in situ spectroscopic methods to estimate the optimal treatment time that provides a biocompatible coating suitable for the realization of the biomimetic approach. The scientific novelty of this research is the justification of the coating thickness diagnostic methods so that the treatment time can be corrected online when the coating achieves the desired properties.

## 2. Materials and Methods

### 2.1. Plasma Electrolytic Oxidation

The PEO process was realized using automated equipment with computer control (USATU, Ufa, Russia) in pulsed bipolar regime under voltage control at positive voltage pulse magnitude U_p_ = 470 V, negative U_n_ = −40 V and duty cycles of the positive pulse of 51%, negative pulse of 26%, with symmetrical pauses were 11.5% of the cycle. The frequency was fixed at 300 Hz. To have a soft start of the PEO process, the pulse voltage was ramped from zero to the setpoint value during 45 s, then the voltage was kept at the setpoint. The process duration was 45, 60, 90, 120, 180, 240, and 300 s. The voltage and current waveforms, root mean square (RMS), and average values were recorded using a data acquisition system operating at f_s_ = 1 MHz; the waveforms T = 200 ms long were recorded every Δt = 1000 ms.

The samples were made of Grade 4 titanium (VSMPO-AVISMA, Verkhnyaya Salda, Russia, ASTM B 348, composition: C—max 0.08%, O—max 0.40%, N—max 0.05%, H—max 0.015%, Fe—max 0.50%, other—max 0.4% total, Ti—balance) in a disk shape with the diameter of 10 mm and thickness of 1 mm. The samples were polished with SiC paper to achieve Ra 0.1 µm. The current was supplied via a wire attached to a small hole at the sample edge; the wire was insulated with an epoxy resin. The electrolyte was 20 g/L Na_3_PO_4_∙12H_2_O; its temperature was kept at 20 ± 2 °C. The electrolyzer was a 6 L plastic tank with a stainless steel coil at the perimeter serving both as a heat exchanger and a counter-electrode. The electrolyte volume was 4 L; a magnetic stirrer was used for the electrolyte circulation.

### 2.2. In Situ Impedance Spectroscopy

In situ impedance spectroscopy was performed in separate PEO experiments with the frequency sweep according to the method described elsewhere [36,43,44]. The equipment details and calibration have been previously discussed [45]. A step scan was used in the range from 20 Hz to 10 kHz. The scan frequencies were evenly distributed in the log scale: 28, 38, 62, 101, 164, 268, 435, 713, 1161, 1845, 3137, 4481, 7843, and 10,457 Hz. Each frequency was applied to the PEO electrolyzer for T = 2 s; therefore, the sweep duration was Δt = 28 s. During each frequency step T, two acquisition frames of T_a_ = 500 ms were recorded at the sampling frequency f_s_ = 1 MHz, which is well above the Nyquist requirement for the highest frequency of 2 × 10,457 Hz. Therefore, temporal and spectral resolutions of the in situ impedance spectra estimates correspond to Δt = 28 s and Δf = 28 Hz, respectively. As shown elsewhere, their product Δt × Δf = 1 [46].

The resultant in situ impedance spectra were processed with the earlier developed software FRAnalysis [41]; the equivalent circuit fitting was performed in ZView (Scribner Associates, Southern Pines, NC, USA).

The PEO process impedance was analyzed as a complex-valued estimate, with slowly time varying modulus |*Z*| and phase angle *θ*:

(1) $$\underline{Z}=Z^{\prime }+j\cdot Z^{\prime \prime }=\left|Z\right|\cdot e^{j\cdot \theta },\;(j=\sqrt{-1}).$$

### 2.3. In Situ Optical Emission Spectroscopy

Optical emission spectroscopy was performed using an AvaSpec-ULS2048-USB2-UA-50 fiberglass spectrometer (Avantes B.V., Apeldoorn, The Netherlands) providing the measurements in the range from 180 to 1100 nm. The optical emission from the PEO tank was delivered by an optical fiber cable, which was housed in the electrolyte in an L-shaped glass tube (USATU, Ufa, Russia) with a quartz window at the end. The distance between the receiving window and the sample was 30 mm. The spectrum integration time was 1 s, which corresponds to the temporal resolution of the method. The spectra were processed using AvaSoft software (Avantes B.V., Apeldoorn, The Netherlands) supplied with the spectrometer. The spectral lines were identified using the NIST atomic spectra database [47]. The optical emission spectroscopy experiments were performed with a simultaneous video recording of the PEO process using samples 10 mm × 40 mm, 1 mm thick.

According to the method in [42], to estimate the coating thickness, the emission intensities of two characteristic peaks were used: *I*_1_—for a component of the electrolyte and *I*_2_—for the substrate material. The coating thickness can be evaluated as:

(2) $$h=k_{1}\cdot (lnI_{1}-lnI_{2})+k_{2},$$

where *k*_1_ and *k*_2_ are the calibration coefficients. In this study, sodium peak Na II at 817.986 nm was used to calculate *I*_1_, and the titanium peak Ti I at 453.501 nm was used for *I*_2_.

### 2.4. Surface Characterization

The coating morphology was studied in top view and in cross-sections using a JSM-6490LV (JEOL, Tokyo, Japan) scanning electron microscope (SEM). The coating porosity and pore size distribution were obtained with ImageJ software in accordance with ASTM E112-10. Furthermore, the pore size distribution was fitted with lognormal distribution, whose mean was interpreted as the mean pore diameter. The coating thickness was measured with a Positector 6000 (Defelsko, Ogdensburg, NY, USA) eddy current gauge with an N-type sensor with an accuracy ±0.1 µm; the thickness was also verified by the analysis of the coating cross-sections. The surface roughness was measured with TR-220 stylus profilometer over the track length 0.5 mm. Ra, Rp, and Rv parameters were analyzed. The phase composition of the coatings was evaluated using X-ray diffractometer (XRD) Ultima IV, (Rigaku, Tokyo, Japan) in Cu-Kα radiation, from 20 to 80 degrees 2θ, using step scan every 0.02 degrees and dwelling time 1 s. The XRD spectra were analyzed using Philips XPert software (Philips, Amsterdam, The Netherlands); a semiquant algorithm was employed for the quantitative analysis.

## 3. Results

### 3.1. Electric Characteristics and Microdischarge Appearance during PEO of Ti

**Figure 1:**
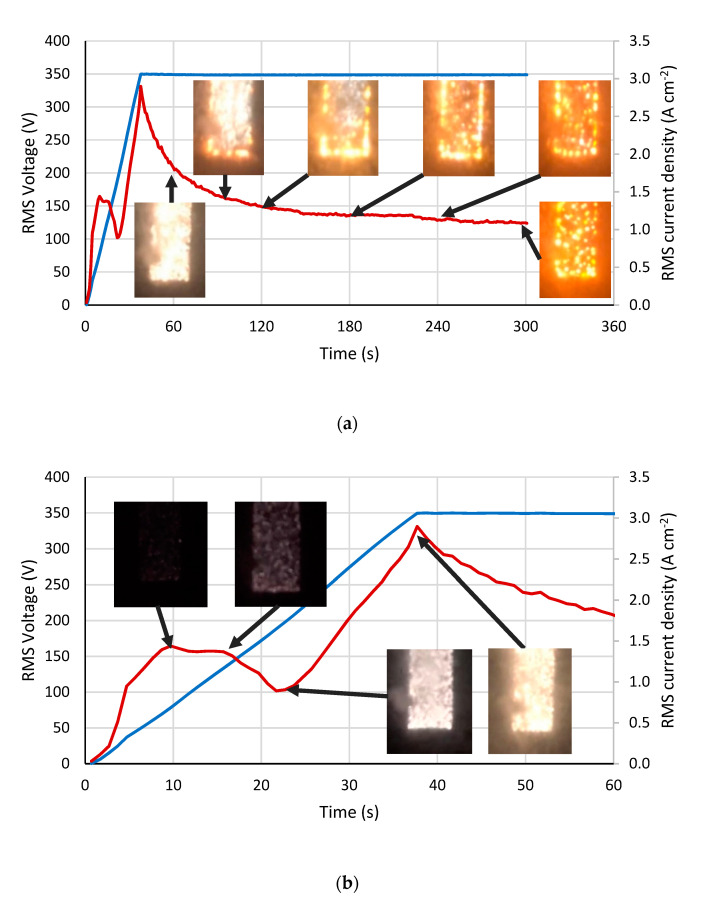
Evolution of RMS values of the voltage (blue) and current density (red), and microdischarges during the PEO process: (**a**) during all the treatment time; (**b**) during the initial stage of the process.

Figure 1 shows the evolution of the RMS voltage and current during the PEO treatment. For each duration of interest, the photo of the microdischarges is shown. The color and the size of the microdischarges change with time: from small white numerous sparks to large yellow sparse “microarcs” (Figure 1a). The initial stages of the PEO process correspond to the voltage ramping during 45 s (Figure 1b). The current density shows complex behavior: growth for the voltage from 0 to 70 V, then a passivation region from 70 to 150 V, followed by the current decrease in the range of voltages from 150 to 200 V, then a sharp increase due to the avalanche microdischarge ignition.

Two competing processes—anodic dissolution and oxide growth—explain the passivation behavior; the second process is prevailing at this stage, and the current stabilizes and even decreases with the voltage growth [26]. This stage is accompanied with the sample chemoluminescence, which gradually highlights the gas bubbles on the surface [48]. The following stage of the microdischarge ignition shows an almost linear current uprise with the voltage growth. The microdischarges significantly increase the conductivity of the system and contribute to the rapid coating growth [10]. By reaching the process steady state after the voltage ramp is over, the current gradually decreases with time; this indicates that the coating gains its thickness, and consequently, electrical resistance. The size of the microdischarges increases as a thicker coating requires more energy for the breakdown of the film.

### 3.2. Evolution of Surface Morphology and Phase Composition during PEO of Ti

**Figure 2:**
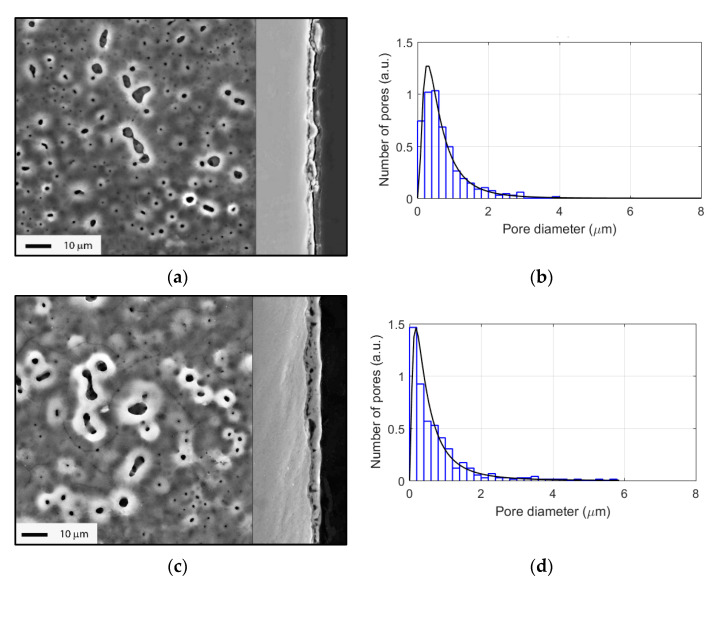

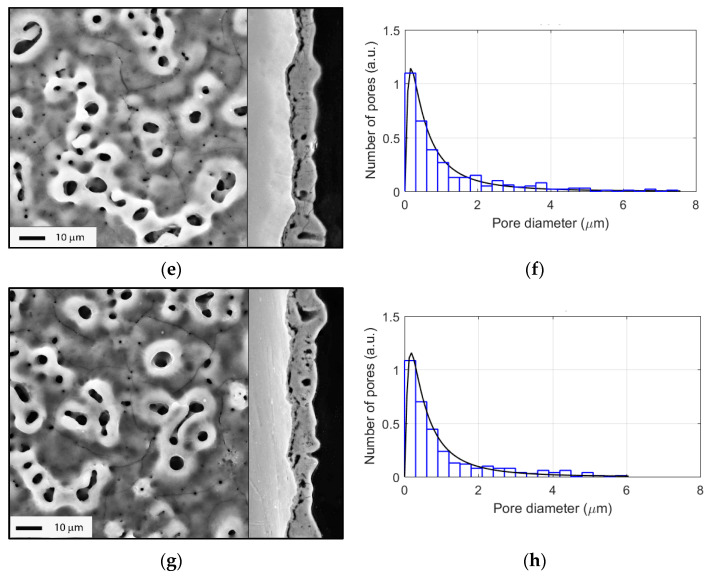
Surface morphology, coating cross-sections, and corresponding pore size distributions (with lognormal distribution fit) for the different PEO treatment times: (**a**,**b**) 45 s; (**c**,**d**) 60 s; (**e**,**f**) 90 s; (**g**,**h**) 120 s.

**Figure 3:**
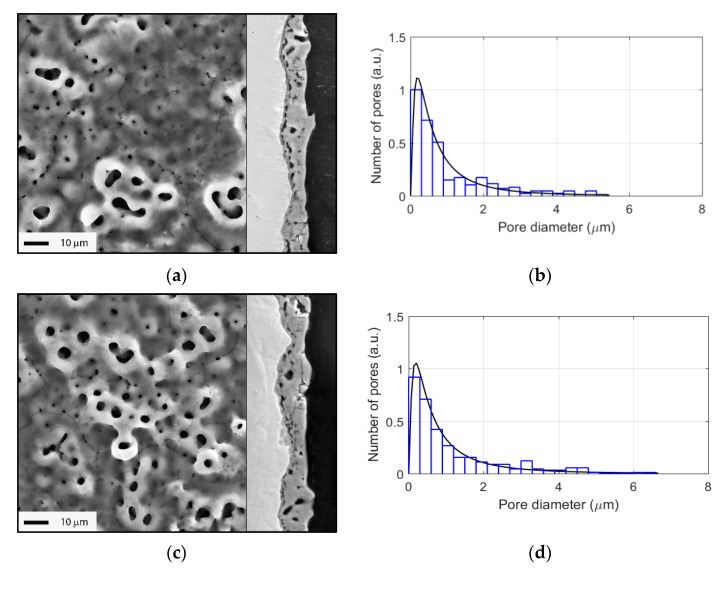

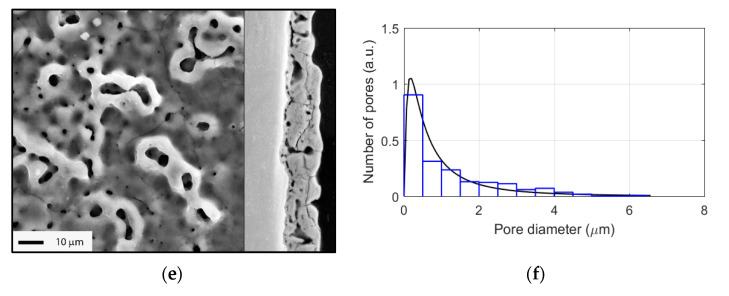
Surface morphology, coating cross-sections, and corresponding pore size distributions (with lognormal distribution fit) for the different PEO treatment times: (**a**,**b**) 180 s; (**c**,**d**) 240 s; (**e**,**f**) 300 s.

The PEO coating morphology in top view and in cross-sections at each duration of interest is shown in Figure 2 and Figure 3. Starting from 45 s, the coating showed a porous morphology. The pore size distribution is depicted in Figure 2 and Figure 3 for each image; the most numerous pores had diameters under 1 µm. The most rapid growth of the coating occurred during the first 120 s of the process (Figure 2). Furthermore, the growth rate decreased, and the coating became larger through the pores and a network of cracks could be seen in the cross-sections.

**Figure 4:**
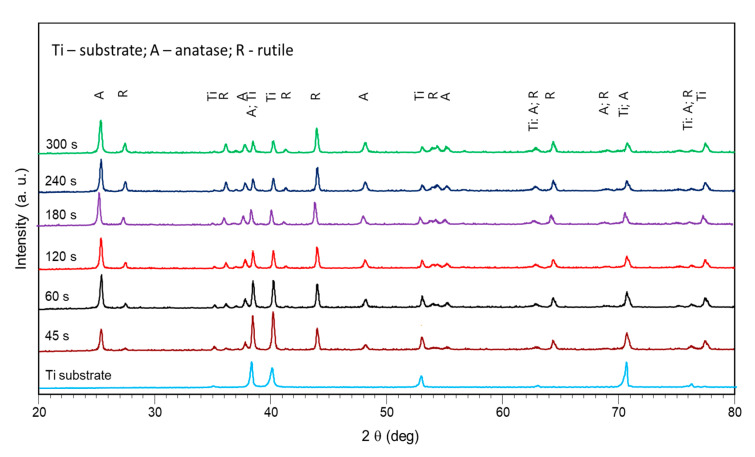
X-ray diffractograms of the coated Ti for the different PEO treatment times.

Figure 4 demonstrates the evolution of the PEO coating phase composition with the treatment time. The XRD revealed the peaks belonging to the Ti substrate, and two phase modifications of titania: anatase and rutile. Thinner coating at 45–60 s showed predominantly anatase peaks (101 at 25.325 deg. 2θ). Rutile peaks (110 at 27.434 deg. 2θ) gradually increased with the treatment time. Since rutile is a higher-temperature phase than anatase, larger microdischarges at the final stages of the PEO provide higher temperatures and the rutile content in the PEO coating increased.

**Figure 5:**
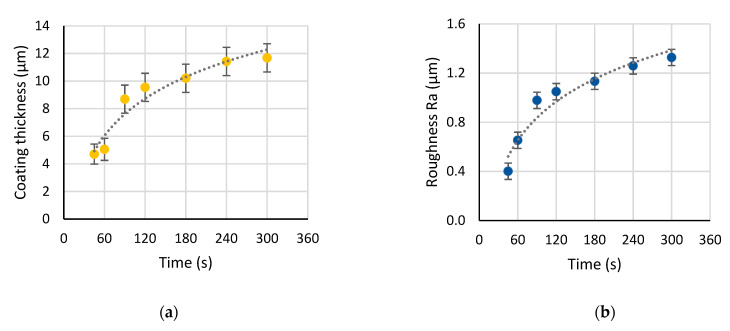

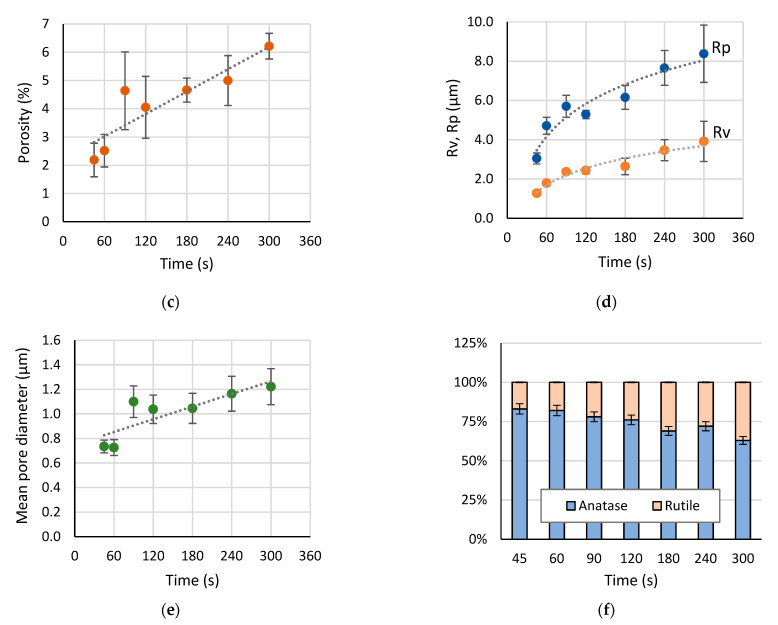
Coating properties vs. PEO treatment times: (**a**) coating thickness; (**b**) surface roughness Ra; (**c**) porosity; (**d**) peak height Rp and valley depth Rv; (**e**) mean pore diameter obtained from the lognormal distribution; (**f**) anatase and rutile content in the coating.

Analysis of the numerical parameters characterizing the coatings is presented in Figure 5. The coating reached the thickness of 10 µm by 120 s of the PEO, showing the growth rate of 5 µm/min; further process was characterized by the rate of 0.5 µm/min, and the coating reached a 12 µm thickness by 300 s (Figure 5a). The surface roughness followed the coating thickness change: Ra rapidly grew from 0.1 to 1.0 µm during 120 s, and then gradually reached 1.3 µm by 300 s (Figure 5b). The peak height Rp and valley depth Rv also increased (Figure 5d); the Rp reached 5 µm by 120 s and a further 8 µm by 300 s. The maximal value depth was 4 µm, which comprises one third of the coating thickness.

The coating porosity almost linearly increased with the PEO treatment time (Figure 5c); starting with 2–3% at 45–60 s, it reached 6% by 300 s. The mean pore diameter obtained from the lognormal distribution increased from 0.7 to 1.2 µm during the treatment (Figure 5e). The content of the anatase linearly decreased with the treatment time (Figure 5f) starting from 83% at 45 s to 63% at 300 s. It should be pointed out that at 90 s of the PEO, all the analyzed parameters showed a deviation toward the increase over the general regularity: the coating thickness, roughness, porosity, and pore diameter were significantly higher than expected. Compared with the current density (Figure 1a), we can expect this to be an effect of the current peak ranging from 30 to 120 s when the current density is higher than the passivation current density value between 10 and 20 s.

### 3.3. Evolution of Optical Emission Spectra during PEO of Ti

**Figure 6:**
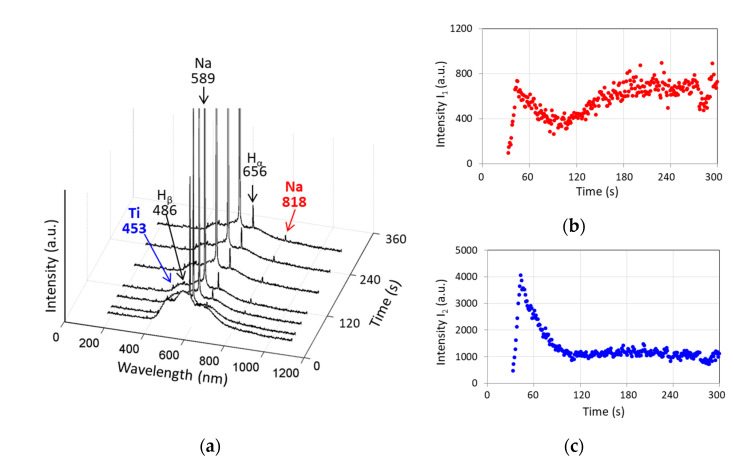
Optical emission spectra of microdischarges during the PEO of Ti: (**a**) overview of the optical emission spectra evolution; (**b**) intensity *I*_1_ for Na II (817.986 nm) representing the component of the electrolyte; (**c**) intensity *I*_2_ for Ti I (453.501 nm) representing the substrate.

Figure 6 shows the evolution of the optical emission spectra of the microdischarges during the PEO of Ti. The spectra comprise characteristic peaks belonging to the elements of the electrolyte and the substrate, and a halo background due to the dissipation in the electrolyte bulk [49]. The strongest Na I peak at 589 nm provides the yellow color of the microdischarges; this peak grew with the treatment time, and its measurements were saturated at the maximal value of the sensor. Therefore, for the application of the method described in Section 2.3, another Na peak was used (Na II at 818 nm) to represent the electrolyte component. The titanium substrate was characterized by a Ti I peak at 453 nm as the strongest one available in the spectrum. The evolution of the intensities of the selected peaks is shown in Figure 6b,c. The electrolyte component line generally grows; between 30 and 90 s, it exhibited a peak following the peak of the current density (Figure 1a). The substrate line decreased after the same peak. Similar spectral peak evolutions were presented elsewhere [50]; however, no conclusions toward the connection with the coating thickness were made in that work.

### 3.4. Evolution of In Situ Impedance Spectra during PEO of Ti

**Figure 7:**
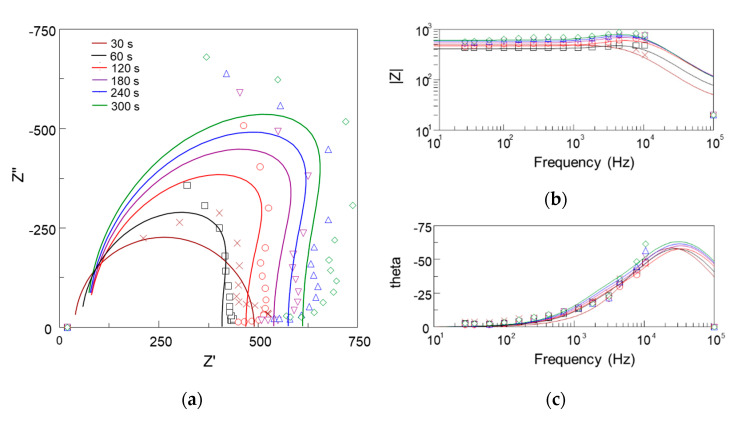
In situ impedance spectra of the PEO process during formation of the coating on Ti with the experimental data and fit results: (**a**) complex plot; (**b**) Bode plot for the impedance modulus; (**c**) Bode plot for the impedance phase angle.

In Figure 7, in situ impedance spectra of the PEO process during formation of the coating on Ti are represented by the experimental data and fit results. Based on earlier results [41,48], the understanding of the equivalent circuits describing the PEO process was enhanced. The complex plots of the in situ impedance spectra had an irregular semi-circular shape appearing in the capacitive half-plane. The approximation toward the highest frequencies corresponded to Rs as the electrolyte solution resistance had a zero phase shift.

**Figure 8:**
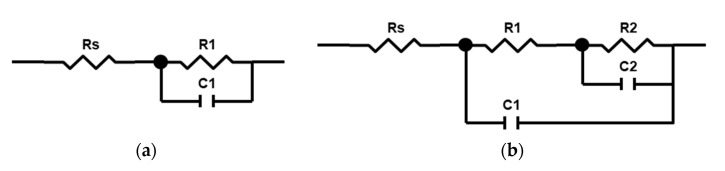
Equivalent circuit for the fitting of the in situ impedance spectra of Ti PEO at different stages: (**a**) 30 s; (**b**) 60–300 s.

The impedance spectra fitting was performed using first- and second-order ladder equivalent circuits (Figure 8). Since the PEO process during the first 30 s comprises mainly anodizing, the first-order Randles circuit was used (Figure 8a) [51]. As the semi-circle in the complex plot bends inside at the lowest frequencies, it is expected to have a RC pair with a negative time constant standing for a negative differential resistance (NDR) of the microdischarges [48]. The fit results are shown in Table 1; for all fits, the electrolyte resistance was the same Rs = 23.5 ± 13.2 Ω.

As seen in Table 1, the values of R2 were negative, and they increased in absolute values. The coating resistance R1 increased with the treatment time from 450 to 1200 Ω·cm^2^. Similar values were obtained earlier using another type of sweep, and with a Voight-type equivalent circuit fitting [41]. The coating capacitance C1 decreased rapidly during the first 120 s and then stabilized at 1.8 × 10^−8^ F·cm^−2^. C2 followed the same pattern.

## 4. Discussion

Analysis of the surface properties during the PEO process shows that the titania PEO coating growth on cp-Ti undergoes the stages of anodic dissolution, passivation, spark microdischarge ignition, microdischarge development, and growth. These stages are reflected as certain regions in the current density plot. This regime results in the porous PEO coating morphology that appears to form within the first 120 s of the treatment when the current density is higher than the passivation value. The coating thickness appears within the range 10–12 µm after 300 s of the treatment. The coating exhibited roughness in the range 0.7–1.4 µm suitable for the osseointegration, as shown elsewhere [52,53]. However, a notable linear growth appeared in the porosity, mean pore diameter, and the rutile content. The coating porosity appeared in the range 4–6% after 120 s; the mean pore diameter was 1.1–1.2 µm throughout 120–300 s of the PEO. The anatase, being the more favorable phase for coating biocompatibility [54], decreased in its content during the process; however, at 120 s, it still comprises over 75% of the coating. As shown elsewhere, rutile must also appear in the coating to provide optimum performance [53].

Analysis of the in situ optical emission spectroscopy results supports the understanding that the substrate line intensities decrease with the PEO treatment time as the coating gains its thickness; this is consistent with other studies of the PEO under voltage control [27], indicating that the substrate emission is blocked by the growing coating. Larger microdischarges provide higher energy for the electrolyte species excitation; therefore, the spectral lines for the electrolyte components grow with the increase in the energy of a single microdischarge for the thicker coating.

Following the understanding of the PEO mechanism where the microdischarges break down the layer having the most dielectric strength, which is the barrier layer on the coating bottom where the microdischarge develops [55], and it is the substrate species that generates the emission first. Therefore, the pore filled with the electrolyte liquid or vapor is ionized after the breakdown of the barrier layer, and the electrolyte species also generate the emission. With the coating growth, the depth of the pores increases, so the intensity of the electrolyte species emission increases. On the other hand, the thickness of the barrier layer does not change with time, and the substrate material emission is masked, and its characteristic line intensity decreases.

**Figure 9:**
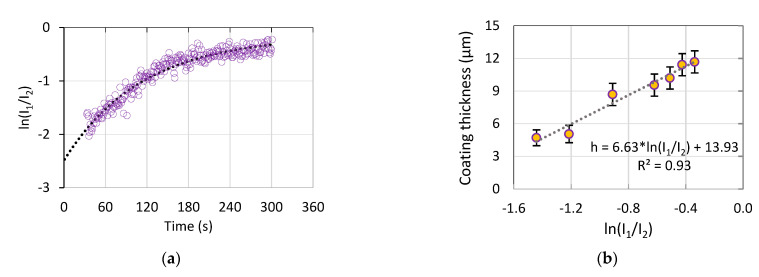

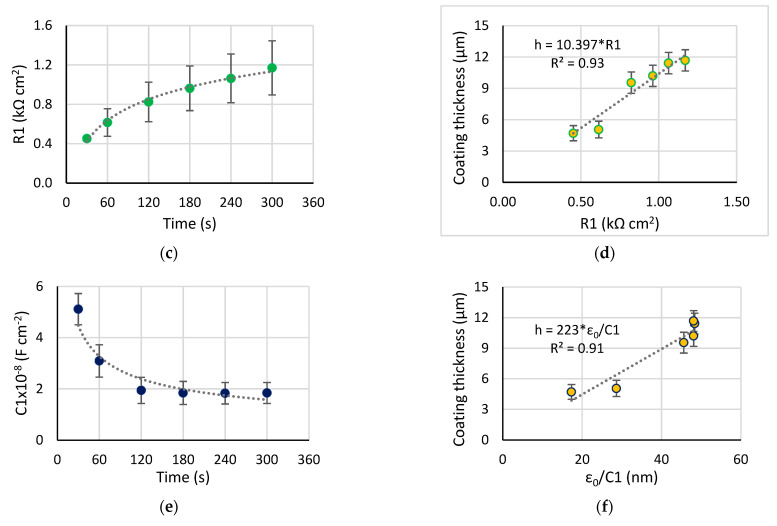
In situ parameters characterizing the Ti PEO, and calibration lines for the coating thickness estimation: (**a**) evolution of ln(*I*_1_/*I*_2_) calculated from the optical emission; (**b**) calibration line for the coating thickness estimation via the optical emission method; (**c**) evolution of R1 calculated from the in situ impedance spectroscopy; (**d**) calibration line for the coating thickness estimation via R1; (**e**) evolution of C1 calculated from the in situ impedance spectroscopy; (**f**) calibration line for the coating thickness estimation via C1.

These regularities support the possibility of indirectly measuring the coating thickness h and other correlated properties (Ra, Rp, Rv) (R^2^ values are in the range 0.92–0.95) via a calibration line with the logarithmic ratio of the characteristic line intensities ln(*I*_1_/*I*_2_) (Figure 9b). It should be pointed out that the temporal resolution of this method corresponds to the spectrum integration time (1 s), and is significantly finer than that of in situ impedance spectroscopy (30 s), therefore, Figure 9a contains a larger number of the experimental points compared to Figure 9c,e. The optical emission characteristics and the coating thickness reached a steady state at 120 s of the treatment; therefore, this duration can be recommended for the termination of the PEO process.

**Figure 10:**
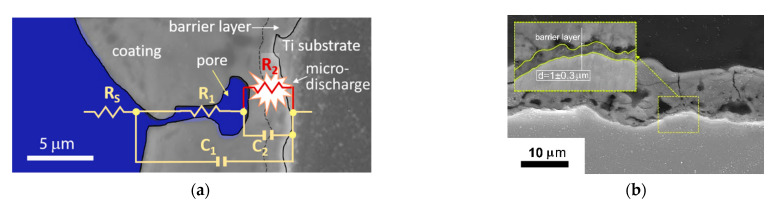
Typical PEO coating cross-section after 180 s of the treatment: (**a**) in situ equivalent circuit corresponding to the PEO process mechanism; (**b**) barrier layer of the PEO coating.

Analysis of the in situ impedance spectroscopy results showed that the proposed equivalent circuit can be a useful tool for the estimation of the process stages and of the coating thickness and other correlated properties. The passivation stage of the process (0–30 s) can be described with a first order Randles circuit comprising the electrolyte resistance, charge transfer resistance R1, and barrier layer capacitance C1. The ladder structure of the circuit, in combination with the negative differential resistance R2, reflect recent understanding of the PEO process mechanism, discussed elsewhere [40]. This supports the hypothesis that the microdischarges do not penetrate the whole thickness of the coating; instead, they break down the inner dense PEO coating layer as having the most dielectric strength. Therefore, the pair R2C2 has an NDR element standing for the microdischarge conductive channel. In contrast, the pore channel in this case is filled with a conductive media—either liquid or vaporized electrolyte [55]—and the pair R1C1 exhibits a positive differential resistance (Figure 10a).

The evolution of the coating resistance R1 was correlated with both the coating thickness and roughness Ra (R^2^ values were 0.93 and 0.96 respectively); their values increased with time at the same rate (Figure 5a,b and Figure 9c,d). The calibration line shows that each kΩ·cm^2^ of R1 stands for 10.4 µm of the resultant coating.

As the dielectric film thickness h is inversely proportional to its capacitance per unit area:

(3) $$h=\frac{\varepsilon \varepsilon_{0}}{C},\;\mathrm{where}\;\varepsilon_{0}=8.85\cdot 10^{-12}\;F\cdot m^{-1},\;$$

the correlation of the coating thickness with *ε*_0_/C1 was also considered (Figure 5a and Figure 9e,f). The capacitance C1 did not change in the time interval from 120 to 300 s of the PEO, indicating the same dielectric properties of the coating. Direct assessment of Equation (3) yielded the value of the coating permittivity *ε* in the range 200–250. This is consistent by the order of magnitude with the estimates for rutile in [56] and for anatase in [57]; however, it was 5-fold higher than the estimate of anodic titania film permittivity *ε* = 45 [58].

The values of C2 appeared in the range of the Cdl evaluated and correlated with the barrier layer thickness via (3) earlier [41]. The steady state value of C2 = 6.5·10^−8^ F·cm^−2^ corresponds to the barrier layer thickness d = 0.6 µm if using anodic titania film permittivity *ε* = 45. This is consistent with a typical coating cross-section (Figure 10b). Consequently, further understanding of the capacitance estimates’ correlation with the coating properties needs separate research.

As a result, the proposed equivalent circuit decomposes the frequency response and provides the elements where physical meaning and evolution correspond to the resultant coating properties (Figure 10b). In this study, the PEO process time of 120 s appeared to be a beneficial trade-off between the coating morphology suitable for the realization of the biomimetic approach, and the energy-consuming process duration.

The analyzed and justified in situ spectroscopic methods help to correct the treatment time in the vicinity of 120 s with a process control and diagnostic system when the coating precisely achieves the desired properties.

## 5. Conclusions

- The plasma electrolytic oxidation in the pulsed bipolar mode under the voltage control, comprising a ramp for the soft start and a steady state regime, provided the active coating growth on cp-Ti for up to 120 s of treatment. After this time, the coating growth rate decreased 10-fold, and up to 300 s, the coating thickness, roughness, and morphology did not change significantly. In the time range from 120 to 300 s, a notable linear growth appeared in the rutile content due to an increase in the power of the microdischarges. Given that for the biocompatibility, the anatase should prevail in the coating composition, the increase in the treatment time is not necessary after 120 s. This coating is suitable for the realization of the biomimetic approach, and it can serve as an inorganic matrix for further introduction of bioactive organic molecules.
- The intensities of the spectral lines of the substrate material, and of the electrolyte species present in the emission generated by the microdischarges changed with the coating growth. Since the spectral line of the substrate material decreases, this supports the contribution to the PEO process mechanism understanding that the breakdown occurs in the barrier layer. Since the electrolyte species’ spectral lines grow, this justifies the ionization of the electrolyte vapor in the deepening coating pore.
- As a result, an optical method was proposed and justified for the coating thickness estimation. The method comprises the log scale ratio of the electrolyte component emission intensity over the substrate material emission intensity; this ratio is highly correlated with the coating thickness and roughness (R^2^ > 0.92), therefore, the coating properties can be estimated during the PEO treatment.
- The in situ impedance spectroscopy helped to evaluate the PEO process frequency response and to propose the underlying equivalent circuit. The microdischarge ignition changed the impedance spectra so that a negative time constant appeared in the system. The impedance spectra fitting with a ladder circuit showed that the negative differential resistance belongs to the inner barrier layer of the coating where the microdischarge starts the breakdown.
- The correlation was established between the resistance R1, 1/C1, and the coating thickness and roughness (R^2^ > 0.93), therefore, the coating properties can be estimated during the PEO treatment with this method. The evolution of the equivalent circuit parameters showed that after 120 s of the PEO, no significant changes appeared, therefore, this time can be recommended for the coating formation.
- Two analyzed and justified in situ spectroscopic methods help to correct the treatment time in the vicinity of 120 s when the coating precisely achieves the required properties by using an appropriately designed process control and diagnostic system.

## Figures and Tables

**Table 1:** Equivalent circuit fit results for the in situ impedance spectroscopy of Ti PEO.

Time (s)	R1 (Ω cm^2^)	C1 (F·cm^−2^·10^−8^)	R2 (Ω cm^2^)	C2 (F·cm^−2^·10^−8^)	Chi-Sqr
30	452.1 ± 24.7	5.11 ± 0.61	-	-	0.399
60	614.9 ± 140.5	3.09 ± 0.63	−260.0 ± 144.1	14.40 ± 18.79	0.333
120	824.0 ± 201.5	1.94 ± 0.51	−427.2 ± 208.5	7.43 ± 8.54	0.381
180	962.1 ± 226.6	1.84 ± 0.45	−496.6 ± 232.2	6.96 ± 7.67	0.366
240	1063.0 ± 247.7	1.83 ± 0.42	−558.2 ± 252.1	6.52 ± 6.91	0.352
300	1170.0 ± 275.3	1.84 ± 0.41	−626.6 ± 278.4	6.03 ± 6.26	0.345

## Data Availability

Data are available upon request from the corresponding author.
